# Benign myoepithelioma of the lung - a case report and review of the literature

**DOI:** 10.1186/1757-1626-3-25

**Published:** 2010-01-13

**Authors:** Jihene Kourda, Olfa Ismail, Bel Hssan Smati, Aida Ayadi, Tarek Kilani, Faouzi El Mezni

**Affiliations:** 1 Department of Pathology, Abderrahman Mami Hospital, Ariana, Tunisia; 2 Department of Cardio-Thoracic Surgery, Abderrahman Mami Hospital, Ariana, Tunisia

## Abstract

**Introduction:**

Benign myoepithelioma is extremely rare in the lung, to the best of our knowledge; only five cases have been reported in the literature.

**Case Report:**

An 18-years woman complained from tiredness and fever during four months. Laboratory findings and fibroscopies were normal. CT of the thorax demonstrated a nodule in the left segment of the Fowler. Left inferior lobectomy was performed comporting a firm nodule of 25 mm, lifting the bronchial mucous membrane. Histologically, there was a proliferation of small cells of a plasmocytoid-type, with a predominantly whorled pattern. No mitotic activity or necrosis was seen in the tumor. Immuhistochemically, the tumor cells positive for smooth muscle actin, vimentine, and S100 protein. They were negatives for cytokeratine, chromogranine and HMB45. The diagnosis of benign myoepithelioma of the lung is so confirmed. The patient recovered well at 6 months follow-up.

**Conclusion:**

Benign myoepithelioma is a rare pulmonary neoplasm distinct from pleomorphic adenoma, which should be considered in the differential diagnosis of lung nodules.

## Introduction

The histological types of primitive "salivary gland-type" tumors arising in the lung are very infrequent. They are essentially represented by the mucoepidermoid carcinoma, the adenoid cystic carcinoma and the pleomorphic adenoma [1].

Benign myoepithelioma is extremely rare in the lung, to the best of our knowledge; only five cases have been reported in the literature [2-5].

## Case Report

An 18-years non-smoker woman complained from tiredness, fever and sweating essentially by night, and during four months. Physical examination was normal except a fever at 39°c. Laboratory findings particularly, complete blood count (CBC) revealed anemia of inflammation; immune-serology was negative for CMV, EBV, toxoplasmosis, HIV, B and C Hepatitis. Biochemical tests were within normal ranges. There was only a major inflammatory disorder especially erythrocyte sedimentation rate (ESR) was elevated > 100 mm/hour, CRP was high in level about 62 U/ml. BK search and IDR reaction were negatives. Digestive and colonic fibroscopy were normal. The initial chest radiograph revealed inter-bronchial centimetric lymph nodes of the left-basal pyramid, without parenchymal lesions. Fiberoptic bronchoscopy was initially normal, with negative core biopsy, aspiration sample and cytology. At an early age, night sweats, the inflammation without obvious port of entry and the fact that Tunisia is an endemic country, the patient was treated as tuberculosis during 2 months. Unfortunately, there was no improvement with persistence of fever and recent weight loss. A new check-up was initiated to absolutely exclude neoplasia. Computed tomography of the thorax finally demonstrated a 25 mm nodule in the left segment of the Fowler, with no extension of the pleural surface. No calcification was seen in the lesion. Fiberoptic bronchoscopy revealed a reddish, hyper-vascularised, gleaming tumor of the Nelson. A carcinoid tumor was suspected. The patient underwent video-assisted thoracoscopic surgery, and a left inferior lobectomy was performed. Gross pathologic findings consisted on a lobectomy measuring 9 × 5 × 4 cm and comporting at 5 mm of bronchial section a firm round nodule. It measured 25 × 20 × 20 mm with well-demarcated margin and lifted the bronchial mucous membrane.

**Figure 1:**
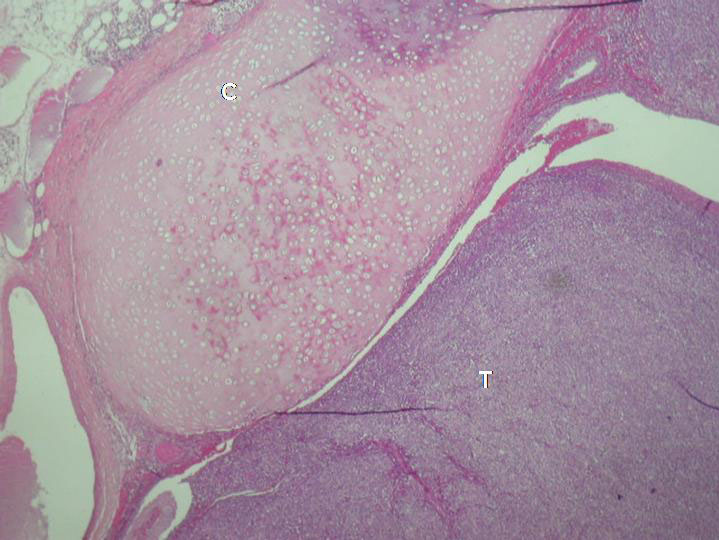
**Endobronchi tumor proliferation (HE ×40)**.

**Figure 2:**
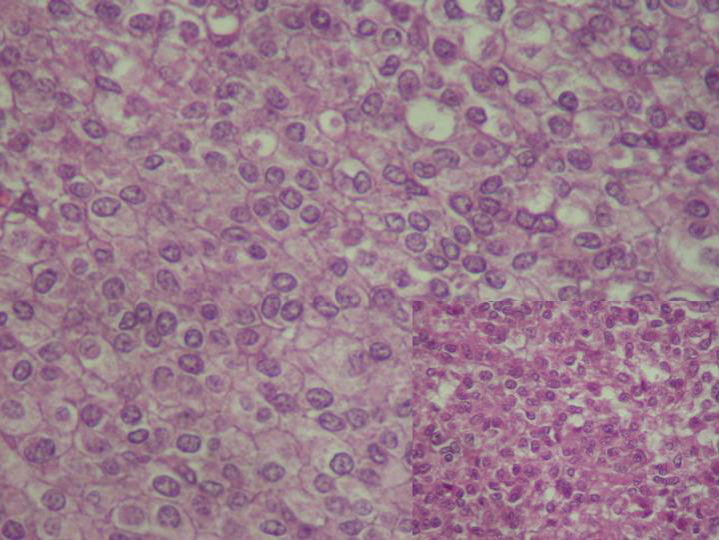
**Small cells proliferation of plasmocytoid-type, in a whorled pattern**. The nuclei showed dispersed chromatin with no mitotic activity (HE ×400).

**Figure 3:**
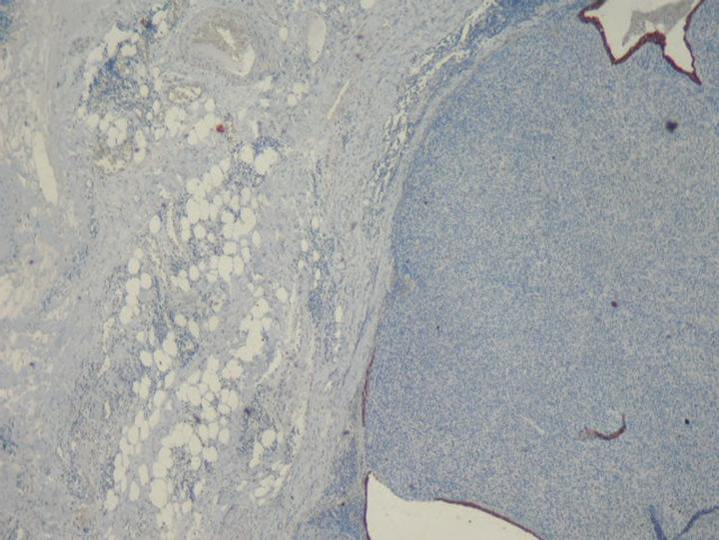
**Immunohistochemestry: Diffuse positivity for Actine smooth muscle**.

**Figure 4:**
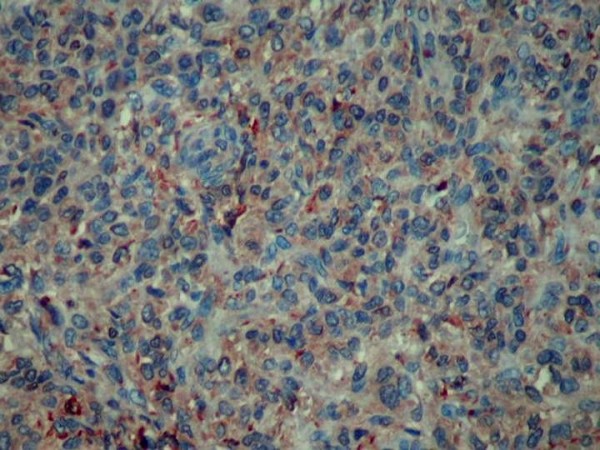
**Tumor cells positive to S100 protein**.

**Figure 5:**
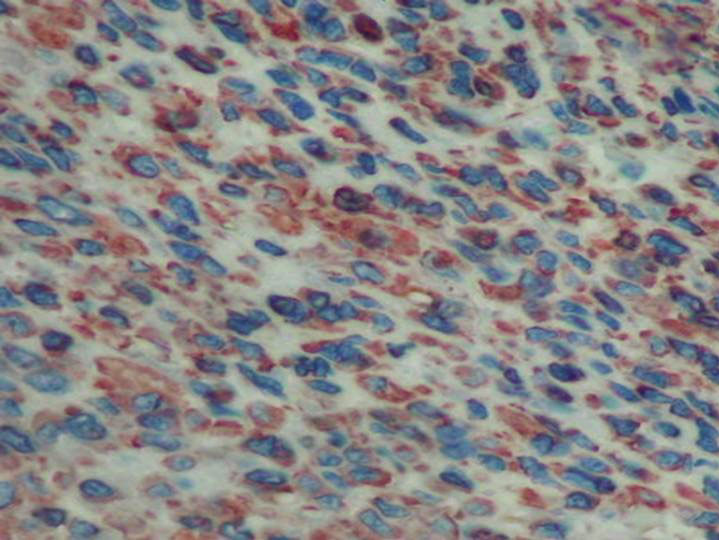
**Tumor cells negatives to cytokeratine**.

Histological findings revealed an endobronchial and submucosal tumor composed of a proliferation of small cells, with a predominantly whorled pattern (Fig 1, 2). There were also areas of focal reticular pattern mixed with pink stroma. Most of the cells appeared as plasmocytoid-type (Fig 2). The nuclei showed dispersed chromatin. A few spindle cells, with cigar-shaped nuclei and abundant eosinophilic cytoplasm. A clear cell changes were focally seen throughout the tumor (Fig 2). Nucleoli were inconspicuous. The benignity of the tumor was confirmed by the absence of mitotic activity, necrosis and hemorrhage. The lack of myxoid or chondroid stroma and glandular structure that eliminates the main differential diagnosis of pleomorphic adenoma. Immunohistochemical stains, including epithelial markers (cytokeratin and epithelial membrane antigen), muscular markers (smooth muscle actin and desmin), neuroendocrine markers (chromogranin and synaptophysin), neural markers (glial fibrillary acidic protein [GFAP] and S100 protein), vascular markers (CD34) and a mesenchymal marker (vimentin), were obtained. The tumor cells were not only positive for smooth muscle actin (Fig 3) and vimentine, but also for S100 protein (Fig 4). Tumor cells were negative for cytokeratine (Fig 5), neuroendocrine markers including chromogranine and synaptophysin as well as for epithelial membrane antigen, desmin, HMB45 and CD34. The MIB1 index was estimated at 1%. All lymph nodes were negative. The diagnosis of benign myoepithelioma of the lung is so confirmed. The patient recovered well following surgery and had no complications at 6 months follow-up.

## Discussion

Myoepithelial cells are usually seen between epithelial cells and basal cells in intercalated ducts and acini of exocrine glands. Myoepithelioma have been described most commonly in salivary gland and accounts for 1% of all tumors developing in the salivary gland. Other sites include soft tissue; breast and skin are well-described entities [6]. In 1987, Strickler et al. [2] reported the first case of a myoepithelioma occurring in the lung. That neoplasm showed histological features identical to those described in myoepitheliomas of major and minor salivary glands. In the lung, myoepithelioma is an extremely rare tumor; only four well-documented cases are reported. In the English literature, Myoepithelioma of the lung have been used as a vague and confusing term to describe a pulmonary lesion of a myoepithelial origin. In fact, several so-called Myoepithelioma were actually malignant tumors [7,8]. Benign myoepthelioma seems to be the best term to define the current lesion to ovoid confusion with malignant myoepithelial lesions in the lung [9]. Histologically, the main differential diagnosis is pleomorphic adenoma or also called mixed tumor. In general, these tumors are characterized morphologically as well-circumscribed lesions exhibiting epithelial and/or myoepithelial elements in variant proportions within a hyalinized to chondromyxiod stroma [10,11]. It has been proposed that this histological complexity in mixed tumors is due to ability of myoepithelial cells to differentiate along many different cell lines. Those tumors, which are composed almost entirely of myoepithelial cells bearing close resemblance to the myoepithelial cells of pleomorphic adenoma and witch are devoid of epithelial ductular differentiation, are referred to as Myoepithelioma [12].

Myoepithelioma is classified by morphologic appearance into four architectural subtypes; solid, myxoid, reticular and mixed, and into five cellular subtypes; spindle, plasmocytoid, epithelial, clear and mixed [10,12]. Lack of macronuclei, multiple nuclei, frequency of mitosis, and high density of nuclear chromatin, assess the benignity. As normal myoepithelial cells, neoplastic myoepithelial cells are generally considered to share both muscular and epithelial characteristics, and have been shown to be immunoreactive for actine smooth muscle (all cases in the litterature) and sometimes for cytokeratine (one case on four). S100 protein is almost positive and expressed in four cases. GFAP is observed in only one case (Table 1). A comparative review of literature emphasizing clinical data's, immuhistochemestry and electron microscopy are detailed in table 1. The MIB1 index was performed only in our case. Ultrastructurally, medium-sized tumor cells present in the cytoplasm, undulating filaments that were located close to the nucleus most consistent with tonofilaments. Myofilaments are also seen arranged in bundles. Both tonofilaments and myofilaments were seen in three cases (Table 1). No electron microscopy was performed in two cases including our case.

**Table 1:** Comparison of all cases of benign Myoepithelioma of the literature

Reference	Location in lung	Size (cm)	Histological type	IHC	Electron microscopy
2	Periphery	3.3	Spindle cells	Actin+ Keratin- PS100+	M

3	Periphery	2.5	Spindle cells	Actin+ Keratin -- PS100+	NA

4	Periphery	1.7	Spindle, plasmocytoid cells	Actin + Kertain+ PS100- GFAP+	T, M

5	Periphery	3	Mixed cellularity	Actin+ Keratin- PS100+	NA

16	endobronchial	7	Mixed cellularity	Actin+ Keratin- PS100+	NA

Current case	endobronchial	2.5	Mixed cellularity	Actin+ Keratin- PS100+	NA

M: myofilaments, T: tonofilaments, NA: not available.

Nuclear expression on P63 was recently described on salivary gland Myoepithelioma [13].

Like any small pulmonary nodule, the myoepitheliomea should be considered as differential diagnosis. Management decisions should not be based on nodule size alone, but patient's age, clinical datas. In fact, previous CT scans, chest radiographs, and other pertinent imaging studies should be obtained for comparison whenever possible, as they may serve to demonstrate either stability or interval growth of the nodule in question. As seen in our case, noncalcified nodules larger than 8 mm diameter can bear a substantial risk of malignancy and should be managed accordingly [14,15]. Depending on the circumstances, follow-up imaging studies or intervention may be appropriate. Primary lung cancer is rare in young patients under 35 years of age (<1% of all cases), and the risks from radiation exposure are greater than in the older population. Like our patient, persons with unexplained fever with certain clinical settings short-term imaging follow-up or intervention may be appropriate. Conservative management is generally appropriate for nodules in very elderly patients or in those with major comorbid disease. Interval growth of any nodule suggests an active process, and further evaluation or intervention should be considered in such cases. Inflammatory disorders, unusual symptoms and endobronchial location were unexpected for Myoepithelioma and make our case unique.

Positron emission tomographic scanning has revolutionized evaluation and staging of benign and malignant pulmonary tumors. Because of the recent advent of this imaging method and because of the rarity of some types of pulmonary nodules, the fluorodeoxyglucose uptake of many such tumors is unknown. In only one patient a positron emission tomographic scan on a benign pulmonary Myoepithelioma was performed for the first time [16]. No fluorodeoxyglucose accumulation was noted in the nodule or mediastinum. This coincides well with the benign histologic findings.

Our case is typically a benign-myoepithelial lesion as the tumour lifted the bronchial mucosa up with no evidence of invasion into it and surrounding lung tissue. No nuclear pleomorphism, hyperchromatism or atypical mitosis was seen. On immunostaining, tumour cells showed positivity for S-100 protein, vimentin and smooth muscle actin.

In salivary gland counterpart, and according to well-documented series, myoepitheliomas are less prone to recur than pleomorphic adenomas. However, others have reported higher recurrence rates. Recurrence is correlated with positive margins at the first excision.

The recommended treatment is complete surgical excision. Benign Myoepithelioma can undergo malignant transformation, especially in long standing tumors with multiple recurrences [17]. This fact is not yet demonstrated in pulmonary Myoepithelioma because of short follow-up.

## Conclusion

In summury, benign Myoepithelioma is a rare pulmonary neoplasm distinct from pleomorphic adenoma, which should be considered in the differential diagnosis of lung nodules, especially when using fine-needle aspiration biopsies. Immunohistochemical stains and electron microscopy, when possible, can accurate diagnosis.

## Consent

Written informed consent could not be obtained because the patient was lost to follow-up. Despite repeated attempts we were unable to trace the patient or her family. We believe this case report holds a worthwhile clinical lesson, which could not be communicated effectively in any other way. Every effort has been made to keep the patient's identity anonymous. We would not expect the patient or her family to object to publication.

## Competing interests

The authors declare that they have no competing interests.

## Authors' contributions

BHS and TK analyzed and interpreted the patient data regarding the pumlmonary disease. They also performed the surgey. JK, OI and AA performed histological examination of the lobectomy. JK and OI collected clinical data's and were the major contributors in writing the manuscript. FEM confirm hisyological diagnosis. All authors read and approved the final manuscript.

## References

[B1] Colby TV Koss MN Travis WD Rosai J Tumors of the lower respiratory tract 1995 Washington, DC: Armed Forces Institute of Pathology

[B2] Strickler JG Hegstrom J Thomas MJ Yousem SA Myoepithelioma of the lung Arch Pathol Lab Med 1987 111 1082 5 2821955

[B3] Cagirrici U Sayiner A Inci I Veral A Myoepithelioma of the lung Eur J Cardiothorac Surg 2000 17 187 9 10.1016/S1010-7940(00)00338-9 10731657

[B4] Veeramachaneni R Gulick J Halldosson AO Van TT Zhang PL Herrera GA Benign Myoepithelioma of the lung. A case report and review of the literature Arch Pathol Lab Med 2001 125 1494 6 11698012 10.5858/2001-125-1494-BMOTL

[B5] El Mezni F Zeddini A Hamzaoui A Ismail O Ghrairi H Ben Miled K Smati B Kilani T Myoépithéliome benin du poumon Rev Pneumol Clin 2004 60 5 282 284 15687912 10.1016/s0761-8417(04)72114-8

[B6] Kilpatrick SE Hitchkock MG Kraus MD Calonje E Flecher CMD Mixed tumors and Myoepithelioma of soft tissue: a clinicopathological study of 19 cases with unifying concept Am Surg Pathol 1997 21 13 22 10.1097/00000478-199701000-00002 8990137

[B7] Higaashimyama M Komdama K Yokoshikawa H Tatsta M Myoepithelioma of the lung: report of two cases and review of the literature Lung cancer 1998 20 47 56 10.1016/S0169-5002(98)00006-3 9699187

[B8] Sekine I Kodama I Yokose I Rare pulmonary tumors-a review of 32 cases Oncology 1998 55 431 434 10.1159/000011891 9732221

[B9] Miura K Harada H Aiba S Myoepithelial carcinoma of the lung arising from bronchial submucosa Am Surg Pathol 2000 24 1300 1304 10.1097/00000478-200009000-00016 10976707

[B10] Dominguez Iglesisas F Fresno Forcelledo F Soler Sanchez T Fernanadez Garcia L Herrero Zapatero A Chondroid syringoma: a histological and immunohistochemical study of 15 cases Histopathology 1990 17 311 317 10.1111/j.1365-2559.1990.tb00734.x 2175294

[B11] Erlandson RA Cordon-Cardo C Higgiins PJ Histogenesis of benign pleomorphic adenoma (mixed tumor) of the major salivary glands: an ultrastructural and immunohistochemical stydu Am J Surg Pathol 1984 8 803 820 10.1097/00000478-198411000-00001 6209992

[B12] Dardick I Myoepithelioma: definitions and diagnostic criteria Ultrastruct Pathol 1995 19 335 345 10.3109/01913129509021906 7483010

[B13] Bilal H Handra-Luca A Bertrand JC Fouret PJ P63 is expressed in basal and myoepithelial cells in human normal and tumoral salivary gland tissue J Histochem Cytochem 2003 51 133 139 12533521 10.1177/002215540305100201

[B14] Tan BB Flaherty KR Kazerooni EA Iannettoni MD American College of Chest Physicians. The solitary pulmonary nodule Chest 2003 123 suppl 1 89S 96S 10.1378/chest.123.1_suppl.89S 12527568

[B15] MacMahon H Austin JH Gamsu G Herold CJ Jett JR Naidich DP Patz EF Jr Swensen SJ Fleischner Society Guidelines for Management of Small Pulmonary Nodules Detected on CT Scans: A Statement from the Fleischner Society Radiology 2005 237 2 395 400 10.1148/radiol.2372041887 16244247

[B16] Dahiya D Endobronchial Myoepithelioma - A Rare Pulmonary Tumour JK Science 2007 9 2 100 101

[B17] Ellis GL Auclair PL Tumor of the Salivary Gland AFIP, Washington 1996 3 57 34

